# MicroRNA miR-24-3p Reduces Apoptosis and Regulates Keap1-Nrf2 Pathway in Mouse Cardiomyocytes Responding to Ischemia/Reperfusion Injury

**DOI:** 10.1155/2018/7042105

**Published:** 2018-12-02

**Authors:** Xu Xiao, Zhigang Lu, Victor Lin, Adam May, Daniel H. Shaw, Zhihao Wang, Briana Che, Kyle Tran, Hongjun Du, Peter X. Shaw

**Affiliations:** ^1^Department of Emergency Medicine, Sichuan Provincial People's Hospital, Chengdu, Sichuan, China; ^2^Department of Ophthalmology and Altman Clinical and Translational Research Institute, University of California San Diego, La Jolla, CA, USA; ^3^Westview High School, San Diego, CA, USA; ^4^Department of Ophthalmology, Xijing Hospital, Xi'an, China

## Abstract

In recent years, microRNAs (miRNAs) have received increasing attention for their role in ischemia/reperfusion injury (I/RI), and many miRNAs have been demonstrated to play a very important role in cardiac I/RI. The miRNA miR-24-3p is a tumor suppressor that regulates multiple tumors; however, it remains unclear whether the expression level of miR-24-3p is altered in cardiac cells under I/RI. In this study, we used mouse primary cardiomyocytes and the H9C2 cardiomyocyte cell line to perform *in vitro* stimulated ischemia/reperfusion (SI/R) and then detected miR-24-3p expression level using quantitative real-time PCR (qRT-PCR). We discovered that the expression of miR-24-3p was significantly increased in cardiomyocytes following SI/R, and that the miR-24-3p level was inversely correlated to the ischemia marker HIF-1a. Furthermore, we transfected cardiomyocytes with miR-24-3p mimic or inhibitor to explore the role of miR-24-3p in cardiomyocyte ischemia/reperfusion injury *in vitro*. We performed flow cytometry to detect the apoptotic rate of H9C2 cardiomyocytes and found that the transfection of miR-24-3p mimic resulted in the decrease of the apoptosis rate of cardiomyocytes after SI/R, whereas the transfection of miR-24-3p inhibitor increased the number of apoptotic cardiomyocytes. These data suggest that the overexpression of miR-24-3p could reduce *in vitro* myocardial cell apoptosis induced by I/R injury. Finally, we applied the dual luciferase reporter gene system to verify whether miR-24-3p targets the Keap1 gene, and found that the luciferase signal intensity from a vector carrying the Keap1 wild-type reporter gene was significantly reduced after transfection with miR-24-3p mimic. The Keap1 protein level was also reduced following the transfection of miR-24-3p. The results from this study suggest a novel function of miR-24-3p in protecting cardiomyocytes from ischemia/reperfusion injury by the activation of the Nrf2-Keap1 pathway.

## 1. Introduction

Cardiac arrest (CA) is one of the most serious acute clinical conditions, requiring immediate rescue to prevent patient death [1, 2]. While cardiopulmonary resuscitation (CPR) is an effective rescue technique, the resulting restoration of spontaneous circulation (ROSC) exposes the body to numerous assaults, including acidosis, electrolyte imbalance, hypoxia, ischemia/reperfusion injury (I/RI), and oxidative stress. Immediately following ROSC, patients often experience severe damage to the heart, brain, and other major organs, leading to systemic inflammatory response syndrome and eventually to multiple organ dysfunction [3–5]. In particular, patients rescued from CA may exhibit myocardial dysfunction. However, the mechanism accounting for the different levels of myocardial dysfunction observed in CA-rescued patients remains poorly understood. Recent studies suggest that the degree of myocardial dysfunction may be associated with the severity of I/RI. Specifically, it has been observed that myocardial reperfusion following a period of ischemia can lead to increased impairment of myocardial structure and function, resulting in decreased cardiac function, myocardial stunning, and malignant arrhythmias [6–10]. Since I/RI poses a major problem in the treatment and recovery of cardiac ischemia, physicians and research scientists have focused on understanding its molecular mechanisms in order to identify potential therapeutic interventions. In our recent study, we demonstrated that in the CA-induced rat model, the post-CPR administration of the antioxidant probucol significantly improved the ROSC rate, hemodynamic parameters, and cardiac function [11]. The probucol also alleviated oxidative stress and prolonged survival time in a dose-dependent manner.

In addition to the administration of antioxidants, one promising approach to alleviate or potentially eliminate the occurrence of I/RI following ROSC is by controlling the microRNA expression. MicroRNA (miRNA) is an endogenous, noncoding single-stranded small RNA molecule consisting of 21–23 bases. miRNAs hybridize with a portion of the untranslated region (UTR) of the messenger RNA (mRNA) from the target gene, directing the assembled ribonucleoprotein complex (miRNP) to either cleave the mRNA or inhibit its translation. Thus, miRNAs function as posttranscriptional regulators of gene expression. It has been observed that one miRNA may regulate hundreds of different target genes or that several different mRNAs may coregulate one target gene [12]. The study found that many diseases' pathogeneses and progressions are closely related to miRNA expression and function. In particular, miRNAs appear to play important regulatory roles in the pathophysiology of heart development, myocardial apoptosis, myocardial remodeling, arrhythmia, heart failure, and cardiac hypertrophy [13–16]. In recent years, several studies have suggested that miRNAs may play an important role in regulating the cardiac response to ROSC and affecting the incidence of cardiac I/RI [17, 18]. For example, a study by Ren et al. demonstrated that miR-320 expression is significantly decreased in a murine model of cardiac I/RI and suggested that miR-320 may regulate rat cardiac I/RI by modulating the expression of the heat shock protein 20 (Hsp20) gene. Knockdown of miR-320 increased Hsp20 protein expression and decreased cardiomyocyte apoptosis induced by cardiac I/RI [19]. In another study, van Rooij et al. found that miRNA-1, miRNA-21, and miRNA-24 were able to reduce infarct size in a non-heat-shock-induced I/RI mouse model [20].

One miRNA whose role in cardiac I/RI has been overlooked is miR-24-3p. In recent years, miR-24-3p as a tumor suppressor [21–24] has been studied in a variety of tumors, such as lung cancer, liver cancer, and nasopharyngeal carcinoma, suggesting that it plays a role in inhibiting tumor cell proliferation and promoting tumor cell apoptosis [25–30]. However, there has been no report regarding the miR-24-3p expression following cardiac I/RI and the role of miR-24-3p in cardiac I/RI. In this study, we sought to determine whether miR-24-3p plays a role in cardiac I/RI and whether the manipulation of miR-24-3p expression can improve the survival of cardiomyocytes, as well as the possible molecular mechanism. In order to test our proposal, we developed two *in vitro* models of cardiac I/RI, in which either C57BL/6 mouse primary cardiomyocytes or H9C2 cardiomyocytes were exposed to hypoxic conditions and subsequently reoxygenated. In each of these models, we determined the apoptotic rate of the cardiomyocytes and assessed the expression of endogenous miR-24-3p in response to both simulation of hypoxia/reoxygenation and transfection of either miR-24-3p mimic or miR-24-3p inhibitor. We also evaluated whether miR-24-3p would affect the apoptosis of cardiomyocytes under stimulated I/RI.

Our previous study also showed that the protective effects of probucol were possibly mediated via regulating the Keap1-Nrf2 system. We demonstrated that the gene expression of Keap1 can be affected by the antioxidant activity of probucol, resulting in improved hemodynamic parameters and cardiac function [11]. This finding leads us to investigate whether miR-24-3p would also target the Keap1 gene and thus affect the Keap1-Nrf2 pathway under cardiac I/RI conditions. In mammals, the Keap1-Nrf2 system is closely associated with the antioxidant response. Under normal physiological conditions, Keap1 and Nrf2 form an inactive complex. However, under increased oxidative stress, Nrf2 dissociates from Keap1, migrates into the cytoplasm, and becomes an active antioxidant factor [31, 32]. A previous study has shown that siRNA knockdown of Keap1 gene expression in human lung fibroblasts changes the antioxidant gene profiling as a novel regulator of the Keap1-Nrf2 pathway [33]. Therefore, verification of Keap1 as one of the target genes for miR-24-3p would suggest a novel mechanistic interplay between miR-24-3p expression and Nrf2 antioxidant activity in modulating myocardial apoptosis and thereby affecting the severity of ischemia/reperfusion injury following cardiac arrest.

## 2. Materials and Methods

### 2.1. Animal and Cell Line

C57BL/6 mice were provided by Shanghai Experimental Animal Center of Chinese Academy of Sciences, with the production license number: SCKK (Shanghai) 0357 and animal license number: Shanghai 22-00653. The animals were housed at our vivarium and fed with regular chow and free to drink purified water. The P2 (2 days postnatal) C57BL/6 mice of both sexes were selected for cell experiments. All mice were housed adaptively for 5 days before the experimental procedure. The food was removed on the eve of the experiment, but the drinking water was not limited. For the cell source, H9C2 cardiomyocyte lines were purchased from the American Type Culture Collection (ATCC). The primary cardiomyocytes were isolated and cultured following the methods described previously [34].

### 2.2. Major Reagents

Cell culture medium (Dulbecco modified Eagle medium, DMEM) for primary cardiomyocytes and H9C2 cardiomyocytes was purchased from HyClone Laboratories, Inc., USA. Fetal bovine serum (FBS) was purchased from Invitrogen, USA. Trypsin was purchased from Gibco Company, USA. Double-resistant solution (penicillin + streptomycin) was purchased from Invitrogen. Goat serum and FITC-labeled goat anti-mouse secondary antibody were purchased from Beijing Zhongjin Jinqiao Biotechnology, China. FITC Annexin V Apoptosis Detection Kits were purchased from BD Biosciences, USA. TaqMan Universal PCR Master Mix and TaqMan microRNA primers were purchased from Applied Biosystems, USA. miR-24-3p mimic, miR-24-3p inhibitor, and RT-U6 primers were purchased from Guangzhou Rui Bo, China. Lipofectamine 2000 transfection reagents were purchased from Invitrogen, USA. The Double-Luciferase Reporter Assay System was purchased from Promega Corporation, USA. The luciferase reporter vectors harboring the wild-type or mutant Keap1 gene promoter or the negative control sequence were purchased from Shanghai Genelec Biochemical Technology Co., Ltd., China. Anti-Keap1 antibody was purchased from R&D Systems, USA.

### 2.3. Isolation of Primary Cardiomyocytes

The neonatal C57BL/6 mice (P2) were euthanized with CO_2_ and the chests were opened. The heart was removed and excised into pieces with a size of about 1 mm^3^. The tissues were digested with trypsin and type II collagenase. The cells were harvested and the cardiomyocytes were collected after differential adherence. After having been seeded on culture plates, cardiomyocytes were incubated in a 37°C incubator. Primary cardiomyocytes were divided into two groups: control group (control group) and simulated ischemia/reperfusion group (SI/R group).

### 2.4. Establishment and Classification of Cardiomyocyte Hypoxia/Reoxygenation Model

When cultured cardiomyocytes reached confluency, the DMEM medium was replaced with serum-free and sugar-free medium. The cells were then placed in a closed 37°C hypoxic incubator (95% N_2_ and 5% CO_2_) with a 95% N_2_ and 5% CO_2_ mixture to vent air at a flow rate of 2 l/min. After hypoxia, the medium was replaced by fresh medium and the incubator was refilled with air containing 5% CO_2_ to establish the cardiomyocyte model of ischemia/reperfusion injury.

### 2.5. Real-Time PCR to Evaluate miR-24-3p Expression

Following the SI/R treatment, the cardiomyocytes were used to extract miRNAs using the mirVana miRNA extraction kit and reverse transcribed to DNA using the TaqMan miRNA reverse transcription kit. The expression of miR-24-3p in myocardium was detected using miR-24-3p miRNA primers and TaqMan Universal PCR Master Mix. The U6 was used as internal reference and the data were expressed as relative to the control.

### 2.6. Real-Time PCR to Evaluate HIF-1a Expression

The SI/R-treated or control cardiomyocytes were used for total RNA extraction for the evaluation of the HIF-1a expression to assay the ischemia status. RT-PCR was performed to quantify the expression level and normalized with GAPDH as an internal control.

### 2.7. Transfection of H9C2 Cardiomyocytes with miR-24-3p Mimic or Inhibitor

When H9C2 cardiomyocytes grew to confluency, they were transfected with miR-24-3p mimic or inhibitor, respectively, using Lipofectamine 2000 according to the manufacturer's manual.

The H9C2 cardiomyocytes were divided into three groups: SI/R group, SI/R + miR-24-3p mimic group, and SI/R + miR-24-3p inhibitor group.

### 2.8. Detection of Myocardial Cell Apoptosis

The apoptosis of cardiomyocytes was detected using flow cytometry for the Annexin V-FITC staining. Briefly, the treated cells were washed with cold PBS and then cells were resuspended in 1x binding buffer and concentration was adjusted to 1 × 10^6^ cells/ml. Then, 100 *μ*l of the solution (1 × 10^5^ cells) was transferred to a 5 ml culture tube and 5 *μ*l of Annexin V-FITC and 5 *μ*l of propidium iodide (PI) were added. The cells were gently vortexed and incubated for 15 min at RT (25°C) in the dark. 400 *μ*l of 1x binding buffer was added to each tube. The cells were analyzed by flow cytometry immediately.

### 2.9. Dual Luciferase Reporter Assay

The Keap1 gene dual luciferase reporter was constructed with either wild-type or mutant promoter. After cotransfection of miR-24-3p mimic or miR-24-3p inhibitor with these reporter vectors along with the renin expression plasmid into H9C2 cells, we detected and analyzed the luciferase activity using the luciferase reporter assay kit. To analyze the data, we designated firefly luciferase activity as M1 and Renilla luciferase activity as M2, and we calculated the ratio of M1 to M2. The relative activity of the reporter gene was compared between each group to determine the target of miRNA.

### 2.10. Western Blot

Experimental cells were collected, homogenized, and lysed with lysis buffer (Cell Signaling Technology, Boston, MA, USA) containing 0.5 mM of phenylmethanesulfonyl fluoride (PMSF) (Sigma-Aldrich, St. Louis, MO, USA). Protein concentration was determined by BCA protein assay (Thermo Fisher Scientific, Grand Island, NY, USA). Samples of 25 *μ*g protein were fractionated by SDS-PAGE in 4–20% gradient Tris-glycine precast gels (Invitrogen) and transferred to a polyvinylidene difluoride (PVDF) membrane (Millipore, Billerica, MA, USA). The membrane was incubated for 1 hour in blocking solution containing 5% nonfat milk powder and 0.1% Tween-20, pH 7.6. This was followed by overnight incubation at 4°C in blocking solution containing rabbit primary antibodies against KEAP1 (D6B12, Cell Signaling Technology, Boston, MA, USA). Subsequently, the labeled proteins were visualized by incubation with a horseradish peroxidase- (HRP-) conjugated anti-goat or rabbit IgG (1 : 2000; Santa Cruz Biotechnology, Inc.) followed by development with a chemiluminescence substrate for HRP (Thermo Fisher Scientific). The images of western blots were captured by GE ImageQuant.

### 2.11. Statistical Analysis

All the molecular biology experiments were performed in triplets, and the results were expressed as mean ± SEM. Data were analyzed by SPSS 18 statistical software. Data comparison between the two groups was performed by unpaired *t*-test. Multiple sets of data were compared using a single factor analysis of variance (ANOVA). *P* < 0.05 was defined as a statistically significant difference.

## 3. Results

### 3.1. The Expression of miR-24-3p Decreased in Primary Cardiomyocytes and H9C2 after SI/R

**Figure 1:**
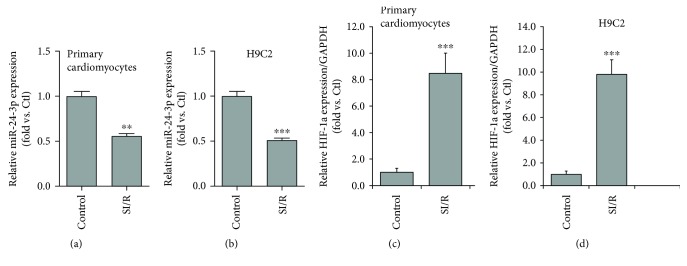
(a) Expression of miR-24-3p in hypoxia/reoxygenation of primary cardiomyocytes. SI/R and control groups, ^∗∗^*P* < 0.01. (b) Expression of miR-24-3p in H9C2 cardiomyocytes after hypoxia/reoxygenation. SI/R compared with the control group, ^∗∗∗^*P* < 0.001. (c) Expression of HIF-1a gene in hypoxia/reoxygenation of primary cardiomyocytes. SI/R and control groups, ^∗∗^*P* < 0.01. (d) Expression of HIF-1a gene in H9C2 cardiomyocytes after hypoxia/reoxygenation. SI/R compared with the control group, ^∗∗∗^*P* < 0.001. Data are expressed as the relative fold change among data sets (*N* = 3) and each was performed in triplicate.

In order to investigate the changes of miR-24-3p expression in primary C57BL/6 cardiomyocytes, we performed qRT-PCR to detect the expression of miR-24-3p in SI/R cardiomyocytes and the control group. The results showed that the expression of miR-24-3p was significantly decreased in neonatal C57BL/6 mouse cardiomyocytes after ischemia/reperfusion compared with that in the control group (Figure 1(a)). Similarly, the expression of miR-24-3p in H9C2 cells after SI/R treatment also showed significant reduction in comparison with the control group after hypoxia for 10 hours and reoxygenation for 2 hours (Figure 1(b)). We have also evaluated the ischemia-associated gene HIF-1a and found that the level of HIF-1a was inversely correlated to miR-24-3p (e.g., increased in the SI/R-treated groups compared to control groups). These results suggest that the miR-24-3p expression level is associated with SI/R cardiomyocyte injury and that potentially increasing its expression may have a protective effect.

### 3.2. Transfection of miR-24-3p Mimic Reduces the Apoptosis Rate of H9C2 Cardiomyocytes under SI/R Injury

**Figure 2:**
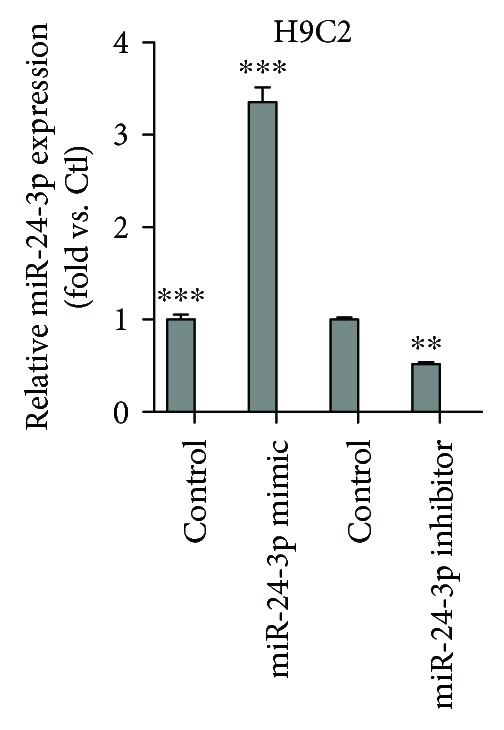
The expression of miR-24-3p following the transfection of miR-24-3p mimic or miR-24-3p inhibitor into H9C2 cardiomyocytes. Data are expressed as the relative fold change in comparison with the untransfected control group (*N* = 3 for each set). ^∗∗^*P* < 0.01 and ^∗∗∗^*P* < 0.001.

**Figure 3:**
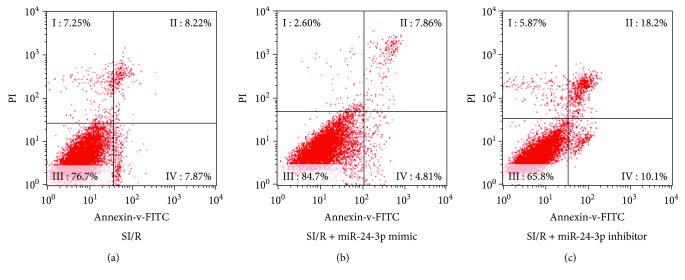
Apoptosis of cardiomyocytes following the transfection of miR-24-3p mimic or miR-24-3p inhibitor into H9C2 cardiomyocytes. A total number of 1 × 10^5^ treated cells were detected by flow cytometry after Annexin V-FITC staining for apoptotic cells. Apoptosis-induced cardiomyocytes were gated into the right upper quadrant (Q2) of each panel while the left bottom quadrant (Q3) contains normal cells and (Q4) cells that were in very early apoptosis according to PI intensity and FSC, as described. (a) H9C2 cells treated only with SI/R, (b) H9C2 cells transfected with miR-24-3p mimic, or (c) H9C2 cells transfected with miR-24-3p inhibitor.

In order to assess whether miR-24-3p affects cardiomyocyte survival after SI/R injury, we first confirmed that the transfection of miR-24-3p mimic in H9C2 cardiomyocytes resulted in a significantly higher level of miR-24-3p in H9C2 cardiomyocytes compared with that of the nontransfection group; while parallelly, the transfection of H9C2 cells with miR-24-3p inhibitor significantly reduced its expression (Figure 2). In the next step, we stained cells with Annexin V-FITC and performed flow cytometry to determine the apoptotic rate of H9C2 cardiomyocytes in each group. Compared with the SI/R group, the apoptosis rate of the SI/R + miR-24-3p mimic group was significantly lower, while the apoptotic rate of cardiomyocytes was significantly increased in the SI/R + miR-24-3p inhibitor group (Figure 3).

The results showed that increasing miR-24-3p could significantly enhance the survival rate of H9C2 cardiomyocytes under SI/R injury (84.7% vs. 76.7% live cells and 6.86% vs. 8.22% apoptotic cells, respectively). On the contrary, the miR-24-3p inhibitor increased the apoptosis rate of H9C2 cardiomyocytes under SI/R injury (65.8% vs. 76.7% live cells and 6.86% vs. 17.4% apoptotic cells, respectively).

### 3.3. miR-24-3p Targets the Keap1 Gene

**Figure 4:**
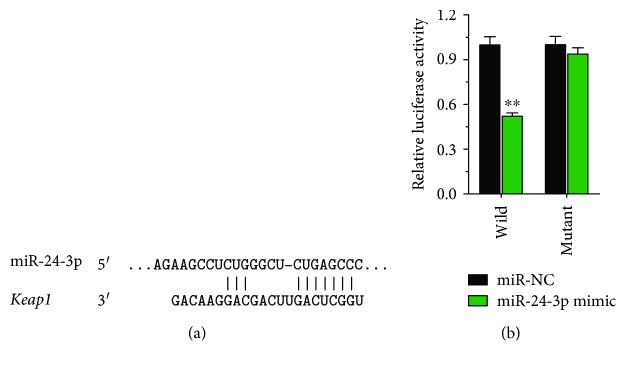
(a) The putative binding site of miR-24-3p is located in the 3′-UTR of Keap1 mRNA. (b) The double luciferase reporter assay system is used to detect the direct interaction of miR-24-3p with the Keap1 3′-UTR. Data show the comparison of the relative luciferase activity of the vector harboring wild-type or mutant Keap1 3′-UTR following the transfection of miR-24-3p mimic (green bars) or nontransfection control (miR-NC) (black bars). ^∗∗^*P* < 0.01.

In order to identify the target of miR-24-3p in cardiomyocyte cells, we screened the putative targeting gene of miR-24-3p using the TargetScan software (http://www.Targetscan.org). We found that the 3′-UTR region of Keap1 contains a binding site for miR-24-3p (Figure 4(a)). To verify whether miR-24-3p regulates the transcriptional activity of Keap1 by targeting the 3′-UTR of Keap1, we constructed a reporter vector containing either wild-type or mutant Keap1 3′-UTR. After cotransfecting miR-24-3p mimic with these reporter vectors into H9C2 cells, we examined the dual luciferase reporter activity and found that transfection of miR-24-3p mimic significantly decreased luciferase signal intensity in cells harboring wild-type Keap1 3′-UTR (Figure 4(b)). In contrast, the vector carrying the Keap1 3′-UTR mutant reporter vector had no significant change in luciferase signal intensity after miR-24-3p mimic transfection. The above results indicate that miR-24-3p can regulate the expression of Keap1 in H9C2 cardiomyocytes, indicating that Keap1 is one of the target genes of miR-24-3p.

**Figure 5:**
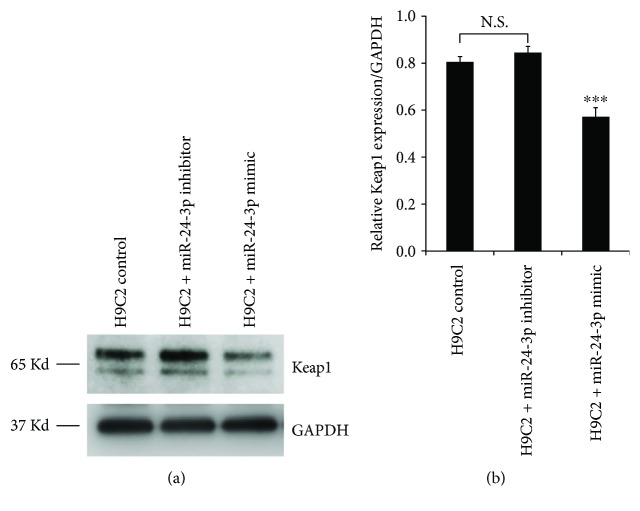
Western blot analysis for Keap1 protein in H9C2 cardiomyocyte control or H9C2 transfected with miR-24-3p inhibitor or miR-24-3p mimic. (a) Representative western blot images. (b) Relative levels of Keap1 protein normalized to GAPDH. *N* = 3; means ± standard deviations are shown. *P* values tested against control are indicated. n.s., nonsignificant.

To directly evaluate the Keap1 protein level in H9C2 cardiomyocytes transfected with either miR-24-3p mimic or miR-24-3p inhibitor, we collected the cells 24 hours after transfection and probed the cell lysate with anti-Keap1. The western blotting showed that the Keap1 level is significantly reduced in H9C2 cells transfected with miR-24-3p mimic in comparison to H9C2 cells without transfection or cells transfected with miR-24-3p inhibitor (Figure 5).

## 4. Discussion

Increasing the recovery rate of CPR from cardiac arrest encounters obstacles in the physiological conditions caused by ischemia, hypoxia, ischemia/reperfusion, and inflammatory response [35–37]. During CPR, myocardial tissue undergoes processes ranging from no perfusion to hypoperfusion to reperfusion injury. During myocardial ischemia/reperfusion injury, a large number of reactive oxygen species (ROS) radicals are released causing lipid peroxidation of biomembranes, which undermine the integrity of cell structure and functions, as well as producing oxidized phospholipids. These oxidation products affect the physiology of cardiomyocytes and the subsequent cellular inflammatory response, apoptosis, and necrosis. In addition, ROS cause cellular DNA and RNA damage, affecting replication and expression which leads to apoptosis and necrosis of cardiomyocytes, myocardial stunning, and reperfusion arrhythmia [38, 39]. The damaged myocardial cells further release oxygen free radicals and intracellular calcium during reperfusion, which enhance mitochondrial damage and the activation of endonucleases, thus further stimulating these processes. As such, administering antioxidants to inhibit myocardial cell apoptosis during CPR and ROSC to reduce myocardial ischemia/reperfusion injury could be a practical therapeutic approach [39–43]. In our previous report, we showed that administration of probucol was capable of significantly increasing ROSC rate and survival time in CA-induced rats after CPR [11].

To investigate gene expression during myocardial I/RI and the functional roles miRNA may play in the process, we turn our attention to miRNA species that are specifically expressed in the myocardium. Studies have found that many miRNAs play an important role in myocardial I/RI. For example, the miR-1 expression was increased in the myocardial I/RI model, and myocardial cell apoptosis was increased by negative regulation of its target genes HSP60 and HSP70 [44]. On the contrary, the expression of miR-133 reduced the apoptosis of myocardial cells through inhibiting the expression of the target proteins of Caspase 9 [45]. In the rat model of I/RI, overexpression of miR-21 in the heart could reduce cardiomyocyte apoptosis and myocardial infarct size, as well as protect the myocardium from ischemia/reperfusion by inhibiting PDCD4 transcription [46].

Since the myocardium injection of miRNA-24 could reduce the infarct size of the non-heat-shock I/RI model in mice [47], we selected miR-24-3p to study its molecular properties related to functions of cardiomyocytes during I/RI using primary mouse cardiomyocytes and H9C2 cardiomyocyte lines. After performing SI/R on both cardiomyocytes, we used qRT-PCR to detect the expression of miR-24-3p and found that its expression level in SI/R-treated cells was significantly increased. We transfected miR-24-3p mimic or miR-24-3p inhibitor into cultured cardiomyocytes to explore the role of miR-24-3p in cardiomyocytes under the SI/R model *in vitro*. We applied flow cytometry to detect the apoptosis rate of H9C2 cardiomyocytes and found that transfection of miR-24-3p mimic decreased the apoptosis rate of cardiomyocytes after SI/R, whereas transfection of miR-24-3p inhibitor increased the apoptosis rate of cardiomyocytes after SI/R. These data suggested that overexpression of miR-24-3p *in vitro* reduced the cardiomyocyte apoptosis induced by ischemia/reperfusion injury.

Since the Keap1-Nrf2 system is closely associated with the antioxidant response [31, 32], our previous study found that Keap1 expression was downregulated significantly, and Nrf2 expression was upregulated significantly in the probucol-treated SI/R rat groups compared to the control animal group. The study indicated that probucol may relieve the oxidative stress in CA-affected rats after CPR through regulating the expression of Keap1 and the Keap1-Nrf2 pathway system, since less Keap1 would shift the equilibrium of Nrf2 after becoming dissociated from Keap1 and migrating into the nuclei to activate the antioxidative factor. In this study, we expanded the previous finding to investigate whether miR-24-3p would directly regulate the Keap1 gene expression.

We first screened the putative targeting gene of miR-24-3p using the TargetScan software and confirmed that the 3′-UTR region of Keap1 contains a binding site for miR-24-3p. Through construction of the dual luciferase reporter gene system with either wild-type or mutant Keap1 3′-UTR, we verified that cotransfection of miR-24-3p mimic with the wild-type Keap1 3′-UTR reporter vector into H9C2 resulted in the decreased dual luciferase reporter activity. In contrast, cotransfection of miR-24-3p with the vector carrying the mutant Keap1 3′-UTR reporter vector had no significant change in luciferase signal intensity. The results provide the evidence that Keap1 may be one of the target genes of miR-24-3p which can regulate the expression of Keap1 in H9C2 cardiomyocytes through binding to its 3′-UTR. We further utilized western blotting to directly evaluate whether the Keap1 protein level in H9C2 cardiomyocytes was affected by transfection with either miR-24-3p mimic or miR-24-3p inhibitor. We found that the Keap1 level is significantly reduced in H9C2 cells transfected with miR-24-3p mimic in comparison to H9C2 cells without transfection or transfected with miR-24-3p inhibitor. Our finding in this report that miR-24-3p downregulates Keap1 adds another dimension of regulating Keap1-Nrf2 pathways in cardiomyocytes, in which microRNA plays a role in limiting Keap1 protein level and can be protective of cardiomyocytes from apoptosis under SI/R injury.

In summary, the present study confirmed that the expression of miR-24-3p was decreased during ischemia/reperfusion injury in mouse hearts. Our *in vitro* transfection experiments show that elevated miR-24-3p expression can rescue the apoptosis of cardiomyocytes during ischemia/reperfusion injury. We also identified that the Keap1-Nrf2 system is one of the potential targets of miR-24-3p, which regulates Keap1 expression through interacting with its 3′-UTR. The data of this study suggest that miR-24-3p may be a potential target for clinical treatment of cardiac ischemia/reperfusion injury.

## Data Availability

The data used to support the findings of this study are included within the article.
